# Georgia Medical Association

**Published:** 1873-03

**Authors:** G. W. Holmes, J. T. Johnson

**Affiliations:** President Georgia Medical Association; Assistant Secretary


### GEORGIA MEDICAL ASSOCIATION.

The next annual meeting of the Georgia Medical Association takes place in the city of Atlanta on Wednesday, the 9th day of April, 1873. We are looking forward to this meeting with unusual interest, and strong hope that it will be the occasion for assembling a very large portion of the medical profession of the State. There is no lack of ability in the ranks of the profession of this State to make the organization alluded to, not only a credit to ourselves, but to the entire country; and as all obstacles to a perfectly harmonious coöperation have been entirely removed, we earnestly appeal to every member of the body and every regular physician in our entire borders to meet us in couucil at the capital of the State at the approaching meeting. We are assured that the medical men of Atlanta, and the citizens of that hospitable city, will be pleased to extend every courtesy to the entire body of the profession.

G. W. Holmes, M.D.,
*President Georgia Medical Association.*

J. T. Johnson, M.D., *Assistant Secretary.*

